# Quantification of dilated deep capillaries in diabetic retinopathy on optical coherence tomography angiography

**DOI:** 10.1038/s41598-023-44848-3

**Published:** 2023-10-19

**Authors:** Noriko Terada, Tomoaki Murakami, Kenji Ishihara, Keiichi Nishikawa, Kentaro Kawai, Akitaka Tsujikawa

**Affiliations:** Department of Ophthalmology and Visual Sciences, Kyoto University Graduate School of Medicine, 54 Shougoin Kawahara-cho, Sakyo-ku, Kyoto 606-8507 Japan

**Keywords:** Retinal diseases, Diagnostic markers

## Abstract

Morphological changes in capillaries are one of major clinical signs in diabetic retinopathy (DR). In this study, we quantified the *dilated deep capillaries* on optical coherence tomography angiography (OCTA) images. Central 3 × 3 mm en face images were obtained using a swept source OCTA device in 105 eyes of 99 patients with DR. Capillaries with a greater diameter in the deep layers were defined as the *dilated deep capillaries*, using stepwise image processing. The relative areas of automatically selected capillaries with a great diameter were calculated as the index of the *dilated deep capillaries*. Most eyes with DR had string-like or dot-like *dilated deep capillaries* in the OCTA images, which appeared to be dilated capillary segments or microaneurysms histologically. They were distributed more densely in the parafovea than in the central sector, while there were no differences between individual quadrants. The index of the *dilated deep capillaries* was higher in eyes with DR than in nondiabetic eyes. The index in the central subfield was modestly associated with visual acuity, diabetic macular edema, and proliferative diabetic retinopathy. The quantitative *dilated deep capillaries* are designated as a biomarker of vision-threatening DR.

## Introduction

Diabetic retinopathy (DR) is a diabetic microangiopathy and often leads to severe vision loss^1^. In healthy retinas, vessels and neurons reciprocally interact via intervening glia, and neurovascular units maintain the blood-retinal barrier^2^. Hyperglycemia stimulates pathological biochemical pathways and the secretion of growth factors and cytokines, and it concomitantly initiates and promotes structural and functional damage in retinal vessels. Histological publications have shown pericyte loss and cell death and proliferation in vascular endothelial cells^3,4^. In particular, trypsin-digested specimens have demonstrated morphological changes in retinal vessels, e.g., microaneurysms and dilated capillary segments^5–7^.

Two-dimensional images of color fundus photography and fluorescein angiography (FA) are ubiquitous imaging modalities and delineate clinically relevant vascular lesions such as microaneurysms, venous beading, intraretinal microvascular abnormalities (IRMAs), and neovascularization^8^. In DR, primary vascular lesions develop in retinal capillaries rather than in arteries or veins, although the color photographs cannot visualize most capillaries. Despite the multilayered structure of retinal vasculature, most deep capillaries are not delineated on FA images^9^. In addition, the source of diffuse fluorescein leakage remains to be elucidated^10^.

Optical coherence tomography angiography (OCTA) allows us to appreciate three-dimensional images of the retinal vasculature including the deep capillary plexus, and to assess diabetic macular ischemia objectively^11–15^. Microaneurysms appear oval or round on fundus photographs and FA images, whereas their morphologies vary in the histological samples after trypsin digestion and OCTA^16–18^. In particular, fusiform microaneurysms and dilated capillary segments, both of which had been described in the histological publications, cannot be distinguished in OCTA images^5–7^. Dilated capillary segments in the deep layer had been clinically neglected lesions because they are not obvious in the two classical modalities.

Recently, dilated capillaries are delineated and automatically detected in deep layers of en face OCTA images^19^. In this study, we prepared the simple method to quantify the index of *dilated deep capillaries* on the en face OCTA images and preliminarily evaluated their associations with vision-threatening DR.

## Methods

### Participants

In this retrospective study, we evaluated 105 consecutive eyes of 99 patients with DR, compared to 30 eyes of 30 nondiabetic subjects. This study was approved by the Kyoto University Graduate School and Faculty of Medicine Ethics Committee and adhered to the tenets of the Declaration of Helsinki. Written informed consent was obtained from each participant.

The eligibility criteria were 1. the eyes of patients with DR for which swept source (SS)-OCTA images of sufficient quality (signal strength index of 8 or more) were acquired and the nondiabetic control, and 2. participants from whom written informed consent was obtained. The exclusion criteria were 1. other chorioretinal diseases, 2. the presence of media opacities interfering with VA or image acquisition, 3. an axial length < 22 mm or > 26 mm, 4. cataract surgery within three months, 5. a history of vitrectomy, 6. prior anti-VEGF treatment, 6. prior ocular steroid treatment, and 7. photocoagulation within 6 months prior to imaging. We further excluded eyes with serous retinal detachment because the outer en face images were not segmented correctly in such eyes.

### Fundus imaging

The best-corrected decimal visual acuity (VA) was converted to the logarithm of the minimum angle of resolution (logMAR). Patients underwent comprehensive ophthalmologic examinations. The diagnosis of international DR severity grades was from the medical records. The axial length was measured using partial coherence interferometry (IOL Master, Carl Zeiss Meditec, Inc., Dublin, CA). Three-dimensional optical coherence tomography (OCT) images were also obtained using Spectralis OCT (Heidelberg Engineering, Heidelberg, Germany), followed by the quantification of the central subfield thickness (CST). Eyes with CST greater than the thresholds (320 μm or 305 μm for male or female patients, respectively) were diagnosed with center-involved diabetic macular edema (DME)^20^.

SS-OCTA images of the 3 × 3 mm square centering on the fovea were acquired using Plex Elite 9000 (Carl Zeiss Meditec, Inc.). It operates at 100,000 A-scans/second using a swept-source tunable laser (a center wavelength between 1040 and 1060 nm). After sequential B-scans were obtained at the same position, flow signals were generated using the optical microangiopathy (OMAG) algorithm. The side-by-side arrangement of B-scans constructs three-dimensional OCTA images. The 300 × 300 A-scans within the nominal 3 × 3 mm square were digitally converted to a 1024 × 1024-pixel array for further analysis. Resultantly, the interscan time was 3 ms.

### Dilated deep capillaries

Each imaging modality casts light on each aspect of the same lesions. Specimens after the trypsin digestion have demonstrated microaneurysms and dilated capillary segments, whereas the latter lesions are not clearly identified on fundus photographs and FA images in DR^5–7^. On *outer* en face OCTA images, dot-like and string-like dilated capillaries were seen but could not be discriminated from each other. We therefore defined capillaries with a great diameter in the *outer* OCTA slab images as *dilated deep capillaries* in this study.

We applied the stepwise image processing to objectively identify the *dilated deep capillaries* and automatically quantify their indices. The steps were as follows: 1. the creation of en face OCTA images in the outer retinal layers, 2. binarization, 3. deletion of fine vessels, and 4. pixel counting of the *dilated deep capillaries* in each sector. We created three patterns of en face OCTA images.

**Figure 1:**
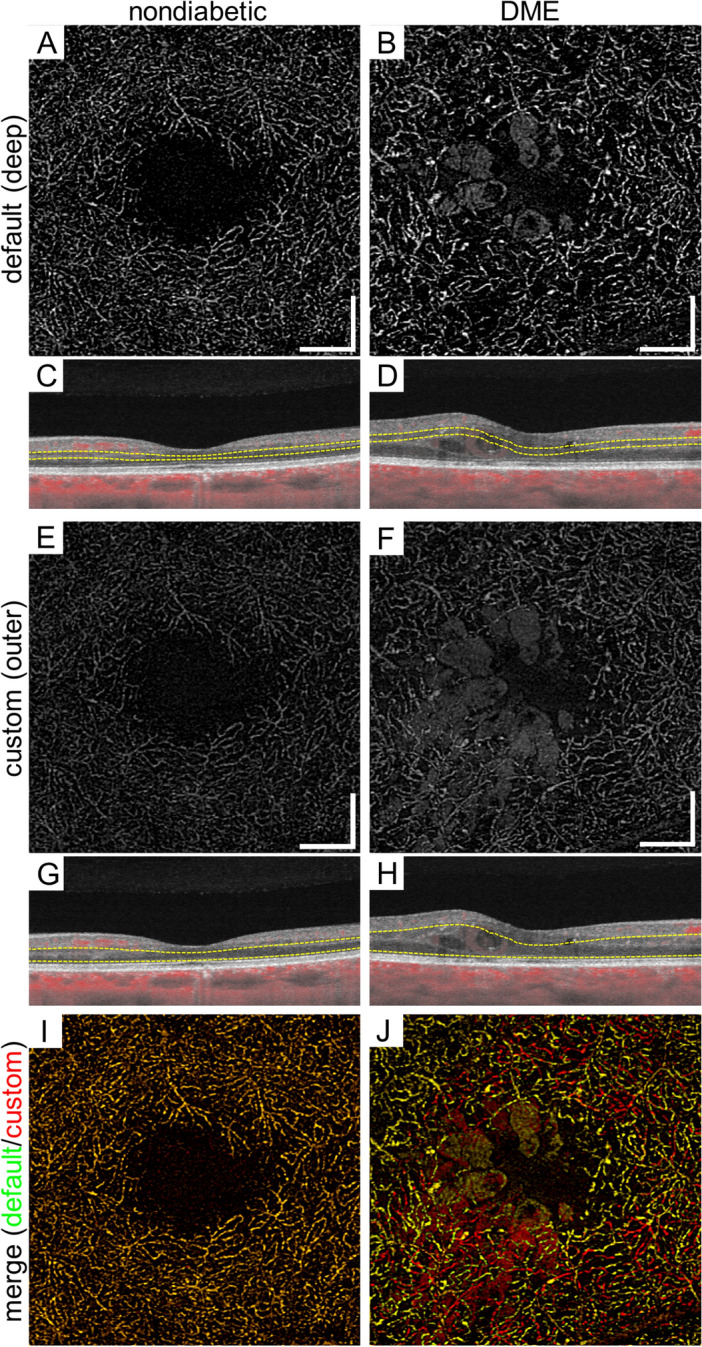
Comparison between the deep en face images by the default setting and the outer images using the custom setting in an eye of a representative nondiabetic control subject and an eye with DME. (**A**, **B**) En face OCTA images of the deep layer using the default setting of the manufacturer’s software. (**C**) The INL is mainly segmented on the B-scan images. (**D**) The vessels in the outer border of the INL is not selected in the edematous areas. (**E**, **F**) The outer en face OCTA images which are created using the custom setting. (**G**, **H**) The corresponding sectional images show the segmentation from the INL to the most parts of the ONL. (**I**, **J**) The merged images demonstrate that deep capillaries are delineated more properly (red) in the outer image than in the deep image in an eye with DME. In contrast, deep capillaries are almost the same in these images in a nondiabetic subject (yellow). (**A**, **C**, **E**, **G**, **I**) A nondiabetic eye. (**B**, **D**, **F**, **H**, **J**) An eye with DME. Scale bar = 0.5 mm.

The default settings of the equipped software made the superficial and deep en-face images. Segmentation error does not allow us to appreciate the correct structure of deep vascular plexuses on the deep slab images created by the manufacturer’s default settings in eyes with DME (Fig. 1). We therefore prepared another slab image from the inner border of the inner nuclear layer (INL) to 70 μm above the retinal pigment epithelium using the custom setting of the same software, which was referred to as the *outer* en face image. Most continuous vessels in the anatomical deep layer were depicted in the *outer* slab image of our setting, whether eyes had DME or not. We therefore selected the *outer* en face image for the further assessment of the *dilated deep capillaries*.

**Figure 2:**
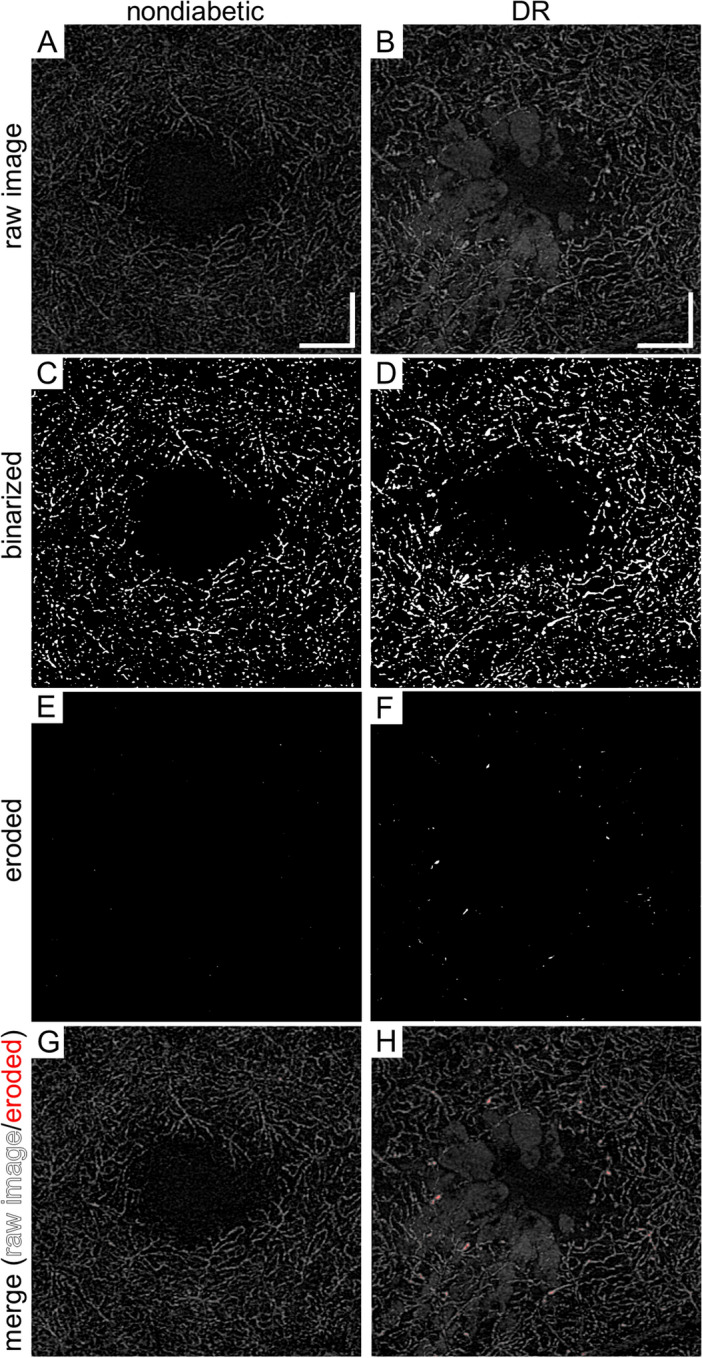
Image processing of the *outer* en face images to characterize *dilated deep capillaries* in an eye of a representative nondiabetic control subject and an eye with DME. (**A**, **B**) Raw OCTA images of the *outer* layers created using the custom setting. (**C**, **D**) Binarized images. (**E**, **F**) Images after the “Erode” function which erases capillaries with a normal diameter. The dilated capillaries of an eye with DME remain visible. (**G**, **H**) The merged images show that the image processing identifies the *dilated deep capillaries* appropriately in an eye with DME. (**A**, **C**, **E**, **G**) A nondiabetic eye. (**B**, **D**, **F**, **H**) An eye with DR. Scale bar = 0.5 mm.

After the automatic adjustment of brightness and contrast, the Auto threshold function (MaxEntropy) of ImageJ (NIH, Bethesda, MD) was applied for the *outer* en face images to produce the binarized images (Fig. 2). Subsequently, the “Erode” function (iteration = 3, number = 1) in ImageJ was used to delete the fine vessels. This function allowed us to delete vessels with a diameter of 6 pixels or less. Most vessels in the deep plexuses had a diameter of 6 pixels or less in the nondiabetic controls. In contrast, both fine and dilated capillaries were seen on the *outer* en face OCTA images in eyes with DR. The Erode function deleted fine capillaries on the OCTA images in both healthy and diabetic eyes and left the *dilated deep capillaries* in eyes with DR. The fourth step was to count the pixels for the *dilated deep capillaries* after image processing in the central 1 mm (central subfield) and four quadrants (superior, nasal, inferior, and temporal) in the parafovea (from 1 to 2.5 mm diameter) after correction for axial length using Bennett’s formula^21^. The percentage of the signals in each sector was calculated as the index of the *dilated deep capillaries*.

### Statistics

The results are expressed as the median (interquartile range [IQR]). The Kruskal–Wallis test with the Bonferroni correction was used for comparisons between groups. Fisher’s exact test was employed to examine the sampling distribution. The receiver operating characteristic (ROC) curve was generated, and the area under the ROC curve (AROC) was calculated to evaluate the DR status–discriminating power of the index of the *deep dilated capillaries* in OCTA images. *P* < 0.05 was considered statistically significant. These statistical analyses were performed using commercial software (PASW Statistics, version 22; SPSS Inc., Chicago, IL).

## Results

### Characteristics of deep dilated capillaries in OCTA images

We evaluated OCTA images of 30 eyes from 30 nondiabetic control subjects and 105 eyes of 99 diabetic patients, and 43 and 41 eyes had center-involved DME and proliferative diabetic retinopathy (PDR), respectively. The characteristics of the participants are shown in Table 1.

**Table 1:** Patient characteristics.

Variables	Nondiabetic subjects	DR
Eyes/patients	30/30	105/99
Age (years)	63 (55–71)	64 (53–71)
Gender (male/female)	18/12	68/31
Hemoglobin A1c (%)	–	7.4 (6.8–8.5)
Duration of diabetes (years)	–	16 (10–23)
Systemic hypertension (present/absent)	16/14	60/39
Dyslipidemia (present/absent)	9/21	41/58
LogMAR	–	0 (− 0.079 to 0.097)
Phakia/pseudophakia	26/4	66/39
International DR severity grade (eyes)	–	
Mild NPDR	–	4
Moderate NPDR	–	48
Severe NPDR	–	12
PDR	–	41
Center-involved DME	–	43
Prior panretinal photocoagulation (eyes)	–	48
Central subfield thickness (μm)	–	293 (269–384)

Data are shown as numbers or the median (interquartile range).

We compared vessels in the deep en face OCTA images to those in the *outer* en face images using the custom setting (Fig. 1). In eyes without DME, both en face images delineated vessels in a similar fashion. In eyes with DME, the deep en face images of the default setting could not depict the whole deep vessels due to the segmentation error. In contrast, the *outer* images with the custom setting allowed us to observe the continuous vascular network of the deep plexuses, and frequently, string-like dilated capillaries were appreciated. In addition, three-dimensional capillary dilation was supposed to be mapped onto two-dimensional en face images. We therefore selected the *outer* en face images in order to evaluate the *dilated deep capillaries*.

**Figure 3:**
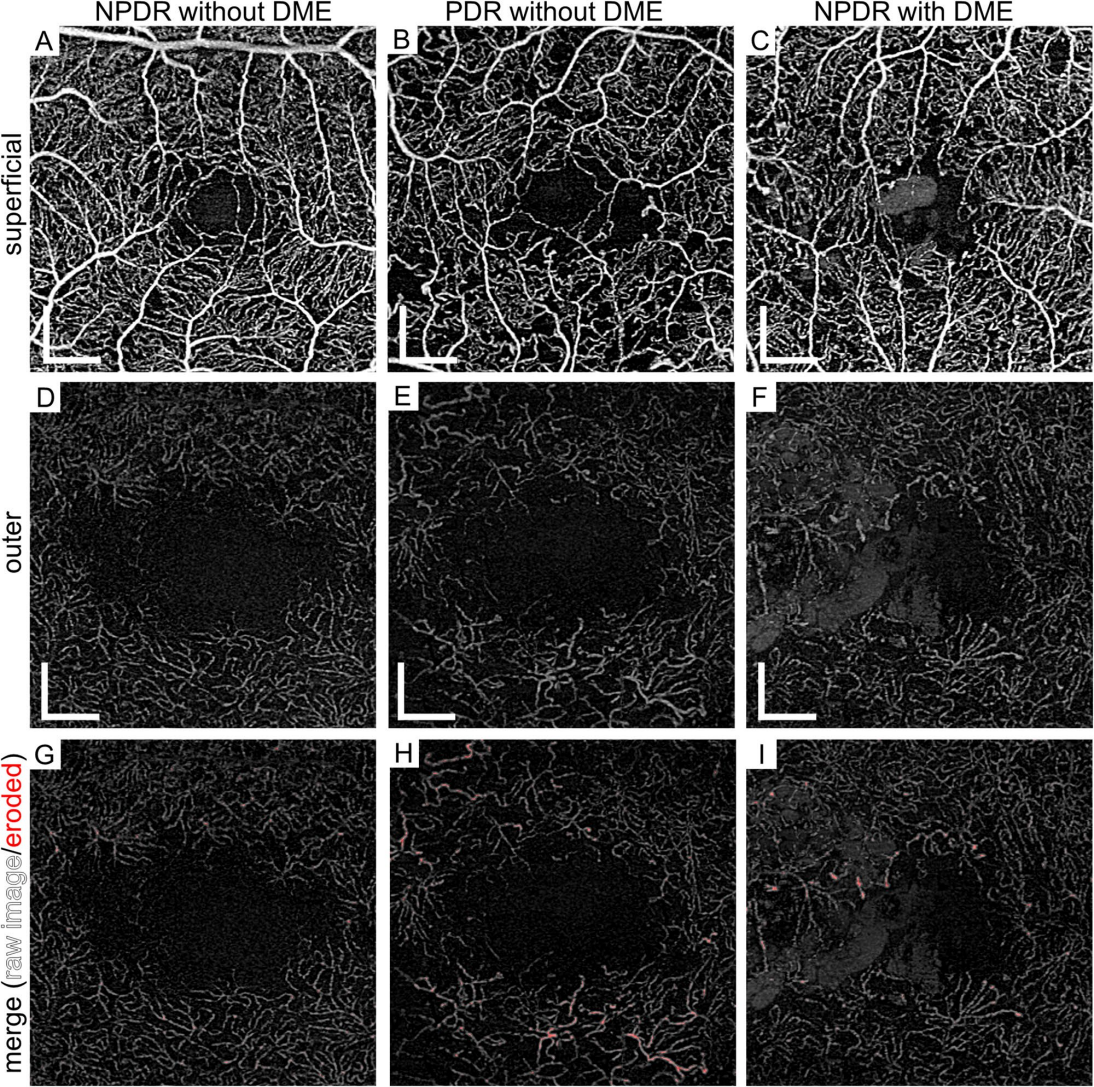
Morphological characteristics of the *dilated deep capillaries* in OCTA images in representative eyes with DR. The superficial (**A**–**C**) and *outer* (**D**–**F**) en face images. (**G**–**I**) The raw and the processed images are merged in the *outer* layer. (**A**, **D**, **G**) An eye with moderate NPDR but not DME. (**B**, **E**, **H**) An eye with PDR but not DME. String-like *dilated deep capillaries* develop. (**C**, **F**, **I**) An eye with moderate NPDR and DME has the dot-like *deep dilated capillaries*. Scale bar = 0.5 mm.

**Figure 4:**
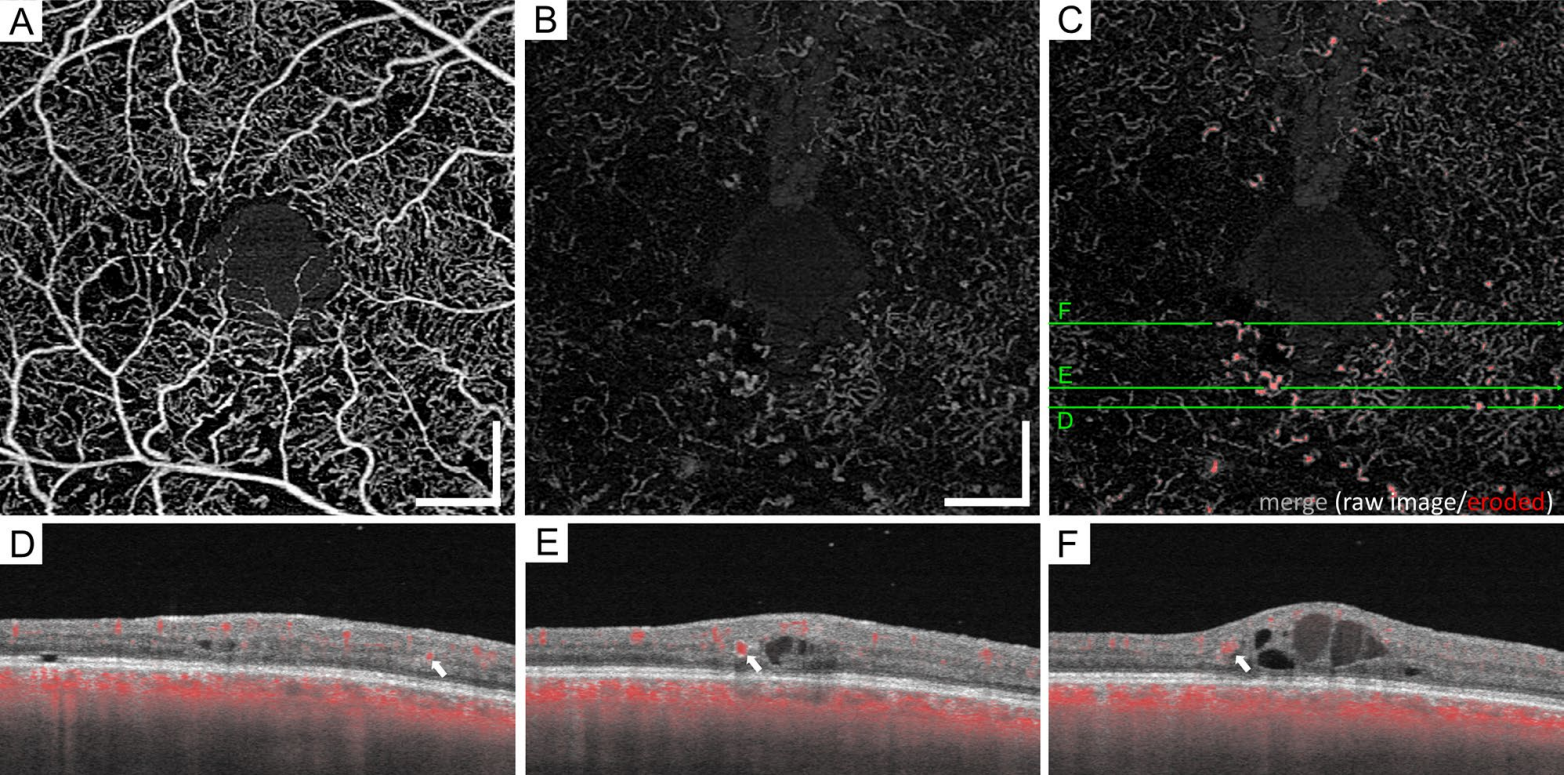
Location of the *dilated deep capillaries* in a representative eye with moderate NPDR and DME. (**A**) In an OCTA image of the superficial layer, the vessels with a greater diameter comprise microaneurysms, arterioles, and venules. The *outer* en-face OCTA image (**B**) and the merged image (**C**) demonstrate that the *dilated deep capillaries* are composed of fusiform or saccular microaneurysms and string-like capillaries. (**D**-**F**) In the sectional images, the *dilated deep capillaries* (white arrows) are delineated mainly in the inner nuclear layer. Scale bar = 0.5 mm.

In the superficial OCTA images, we observed dot-like dilated capillaries rather than string-like capillaries, whereas both vascular lesions were often delineated in the *outer* en face images (Fig. 3). Dot-like and string-like *dilated deep capillaries* might correspond to microaneurysms and dilated capillary segments in the histological publications, respectively^5–7^. However, they could not be discriminated from each other. Typical dot-like dilated capillaries showed multiple morphologies, e.g., fusiform and saccular. In the B-scan images, most of them were located mainly within the INL, whether they originated from the superficial or deep plexuses (Fig. 4). It is consistent to the location of microaneurysms shown in structural OCT images^22^.

### Clinical relevance of the deep dilated capillaries

**Figure 5:**
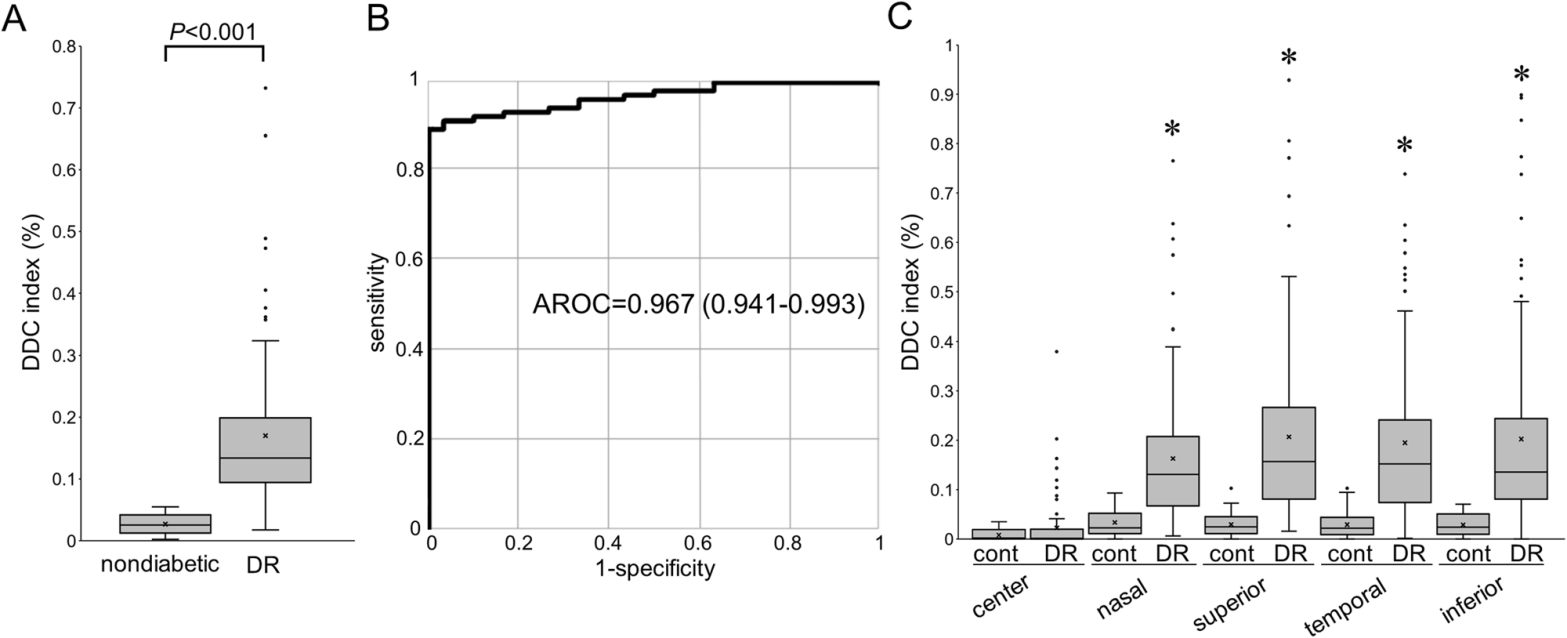
The quantified index of the *deep dilated capillaries* (DDC) in eyes with DR. (**A**) The index of the *deep dilated capillaries* within the central 2.5 mm circle is significantly higher in 105 eyes with DR than in 30 nondiabetic eyes. (**B**) The ROC analysis to discriminate DR from the nondiabetic control reveals a large AROC. (**C**) The indices are higher in the sectors of the parafovea than in the central subfield. *P < 0.001 vs. center.

The indices of the *dilated deep capillaries* were significantly higher in eyes with DR (0.133% [0.095–0.195]) than in those of control subjects (0.042% [0.018–0.048]) (Fig. 5A). The ROC analysis revealed that the index had the significant power to discriminate DR from the nondiabetic control (Fig. 5B). The nasal (0.133% [0.068–0.210]), superior (0.157% [0.081–0.260]), temporal (0.152% [0.074–0.239]), and inferior (0.138% [0.081–0.244]) sectors of the parafovea had higher indices than the central subfield (0.001% [0.000–0.018]) (Fig. 5C), although we did not find significant differences in the *dilated deep capillaries* between quadrants.

**Figure 6:**
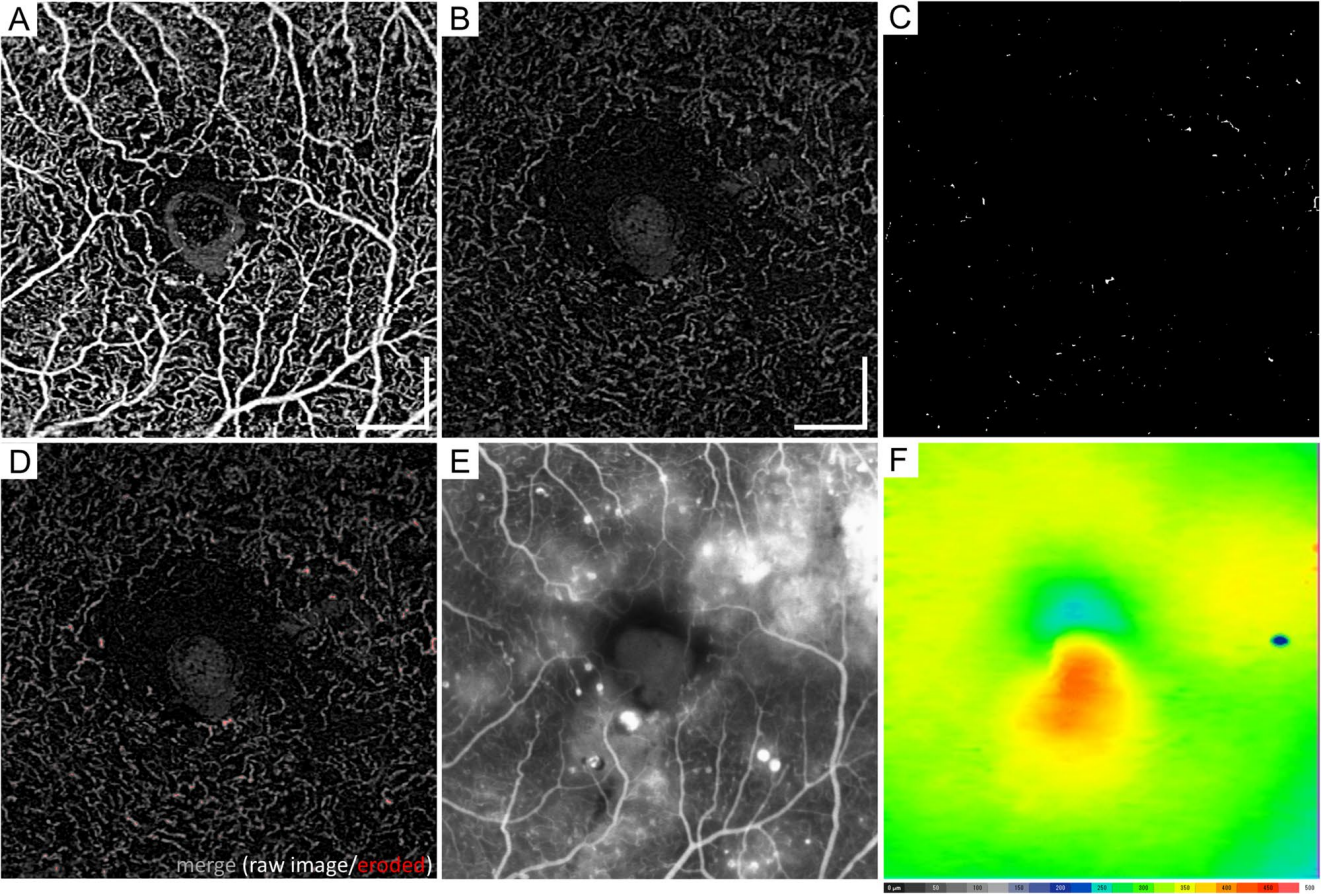
Comparisons between OCTA and FA images in a representative eye with moderate NPDR and DME. (**A**) The superficial en face OCTA image. The *outer* en face OCTA image (**B**), the corresponding processed image (**C**), and the merged image (**D**) show that the *dilated deep capillaries* are delineated in the superotemporal and inferior areas of the parafovea. (**E**) The corresponding FA images in the late phases. Most of the *dilated deep capillaries* in the parafovea on the OCTA image do not correspond to vascular lesions in the FA image. Diffuse fluorescein leakage (**E**) and mild retinal thickening on the two-dimensional OCT map (**F**) develop in these areas. Scale bar = 0.5 mm.

**Figure 7:**
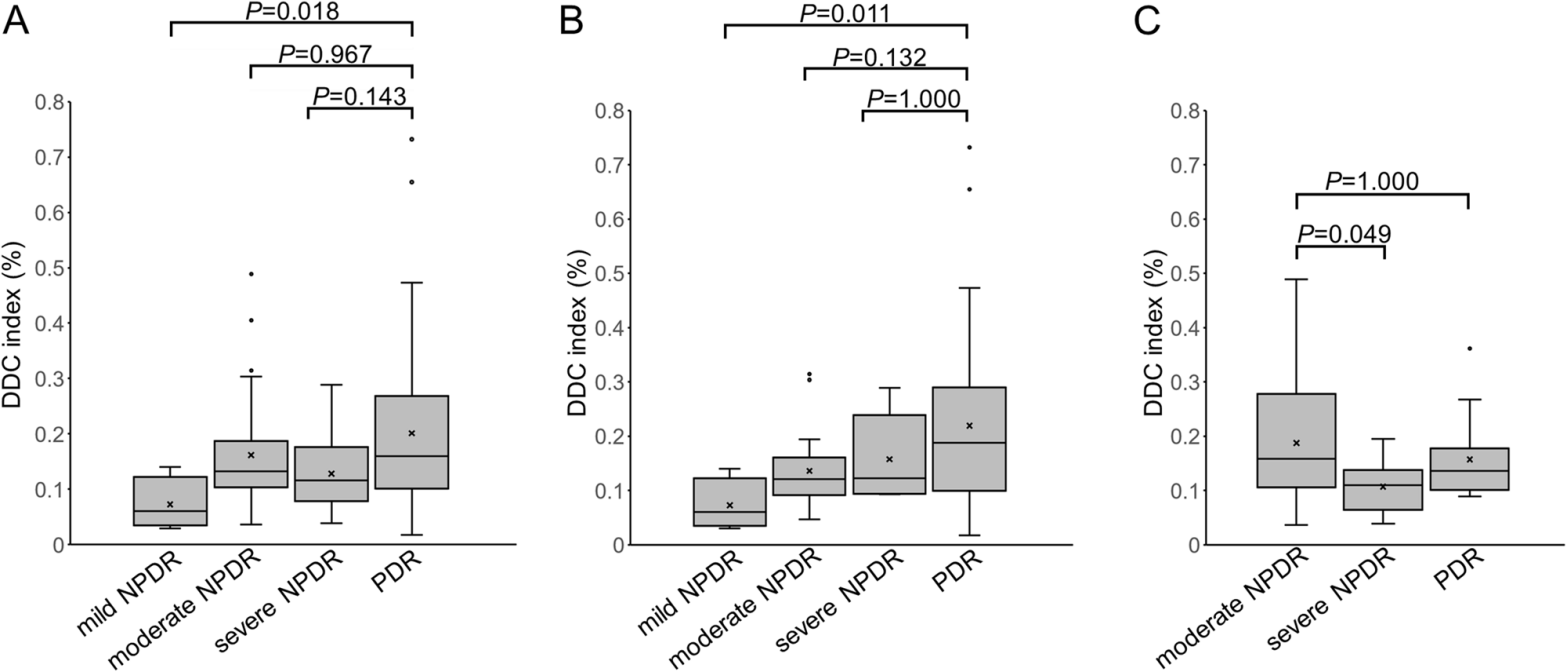
The index of the *dilated deep capillaries* (DDC) in each DR severity grade. The indices of the *dilated deep capillaries* gradually increase according to the DR severity grade in all 105 eyes with DR (**A**) and in eyes without DME (**B**). Conversely, eyes with moderate NPDR have higher index than those with severe NPDR in the DME group (**C**).

We investigated the clinical relevance of the index of the *dilated deep capillaries* in DR. Twenty-seven (48.2%) of 56 eyes with more than zero of the indices in the central subfield had poor vision (logMAR < 0) more frequently than eyes with zero of the indices (13 [26.5%] of 49 eyes; *P* = 0.027). We therefore investigated the relationship between the index of the *dilated deep capillaries* and vision-threatening DR. In the nonproliferative diabetic retinopathy (NPDR) groups, eyes with DME had a higher index in the central subfield and the temporal quadrant in the parafovea than eyes without DME (Table 2 and Fig. 6). The indices were higher in eyes with PDR than in those with mild NPDR in the overall group or the no DME group (Fig. 7A,B), although eyes with moderate NPDR had higher indices than those with severe NPDR in the DME group (Fig. 7C).

**Table 2:** The index of the *Deep Dilated Capillaries* in NPDR eyes with or without center-involving DME.

	NPDR without DME (n = 33)	NPDR with DME (n = 31)	*P*-value
All sectors	0.119% (0.091—0.160)	0.133% (0.105–0.199)	0.110
Center	0.000% (0.000–0.005)	0.009% (0.000–0.026)	0.033
Parafovea			
Nasal	0.138% (0.076–0.181)	0.092% (0.053–0.194)	0.856
Superior	0.146% (0.073–0.200)	0.157% (0.086–0.237)	0.359
Temporal	0.095% (0.070–0.152)	0.164% (0.064–0.283)	0.024
Inferior	0.134% (0.097–0.183)	0.131% (0.067–0.211)	0.245

Data are shown as the median (interquartile range).

## Discussion

String-like capillary dilation was often delineated in the deep vascular plexus in OCTA images, which could not be clearly discriminated from microaneurysms. We therefore defined both vascular lesions as the *dilated deep capillaries* on the *outer* en face OCTA images in DR in the current study. We demonstrated the simple image processing which allowed us to quantify them objectively. The index was higher in eyes with DR than in those with nondiabetic controls. In addition, eyes with PDR had a higher index than those with mild NPDR. The *dilated deep capillaries* were more frequently delineated in eyes with DME than in those without DME in the NPDR group. These data suggest the clinical relevance and may promote our understanding of the pathogenesis of capillary lesions in vision-threatening DR.

Microaneurysms appear to be round or oval on color fundus photographs and FA images^7,23,24^. In contrast, saccular or fusiform microaneurysms and dilated capillary segments are not discriminated from each other in trypsin-digested specimens of DR patients^5,6^. In other words, they seem to be the continuum morphologically. On *outer* en face OCTA images, microaneurysms cannot be clearly determined, which is similar to the histological findings rather than the FA findings^16–18^. This encouraged us to define both string-like and dot-like capillaries with a greater diameter as *dilated deep capillaries* on the *outer* en face OCTA images. We speculated that such OCTA lesions may correspond to the typical microaneurysms and dilated capillary segments in histological publications^5,6^. String-like *dilated deep capillaries* were morphologically similar to IRMAs in the superficial layer, although the layers where these lesions developed were not consistent.

We further evaluated the clinical relevance of the *dilated deep capillaries*. A higher AROC of the quantitative index to discriminate DR from the nondiabetic control suggests the diagnostic significance. It may be consistent with the subjective and qualitative analysis of dilated capillaries in the deep layer and the relevance of diabetic microaneurysms. This finding may be consistent with the previous publications regarding the relevance of diabetic microaneurysms and the qualitative analysis of dilated capillaries in the deep layer^25,26^. The index of *dilated deep capillaries* might be more highly reproducible than the subjective assessment of microaneurysms on OCTA images. Microaneurysms are generally an indicator of DR progression^8,27^. The quantitative parameters are generally feasible for the longitudinal study, and future study should describe the course of the *dilated deep capillaries* and elucidate whether the index can also predict the exacerbation or improvement of DR severity and DME.

Some eyes with PDR had the highest index of the *dilated deep capillaries*. This is consistent with the fact that typical angiogenesis and capillary dilation share the common biological processes, e.g., endothelial proliferation, loss of pericyte coverage, and the remodeling of the basement membrane^5,6,28,29^. However, future study should elucidate the molecular mechanisms which determine angiogenesis from superficial vessels into the vitreous or capillary dilation in deep vessels.

After the exclusion of PDR cases, eyes with DME had higher indices of the *dilated deep capillaries* in the central and temporal sectors. As in the case of microaneurysms with focal fluorescein leakage, the blood-retinal barrier might be broken in some of the *dilated deep capillaries*, which contributes to diffuse fluorescein leakage or the development of DME^10^. VEGF derived from ischemic retinas might induce both capillary dilation and vascular hyperpermeability^29–32^. In addition, it is enigmatic that eyes with moderate NPDR had a higher index of the *deep dilated capillaries* than those with severe NPDR in the DME group. In eyes with moderate NPDR, the pathogenesis in center-involved DME may depend on local VEGF or dilated capillaries with vascular permeability in the central areas. In eyes with severe NPDR or PDR, globally increased VEGF may induce permeability in the macula before capillary dilation there^30^. Another explanation is that the small sample size might lead to these inconsistent results accidentally.

We have to confess that the index of *dilated deep capillaries* depends on the artifacts in the image acquisition and the methodologies of image processing in the current study^33–35^. In addition, OCTA could not tell us the exact diameter of capillaries. The lateral resolution of OCTA images is not sufficient to depict the exact diameters of healthy and pathological capillaries, and the retinal capillaries in OCTA images are wider than those in the histological publications^36^. However, vessel diameters in OCTA and other imaging modalities decrease proportionally, as they run towards the periphery. It may allow us to hypothesize that the vessel diameters on OCTA images are strictly correlated with histological ones. Another point is that the index is not an absolute value but a relative value of the dilated capillaries.

The *outer* en face images contain both intermediate and deep vascular plexuses, which can be divided in projection resolved OCTA. Future study may elucidate the vascular lesions for specific plexus layers. This image processing cannot completely delete the vessels at the branching points, which were sometimes dilated. In addition, flow signals are temporarily absent in diabetic microaneurysms^18,35^. To exclude the signals of the suspended scattering particles in motion (SSPiM), we used the global thresholding to prepare binary images of the retinal vessels^37^. This function omits the faint flow signals in capillaries. It suggests that we did not assess all the *dilated deep capillaries* on the binary images in this study. Additionally, SSPiM per se may influence the signal levels of retinal vessels and concomitant quantification of the index of *dilated deep capillaries*.

There are several limitations in this retrospective study, particularly selection bias and specific methods for image acquisition and processing. The ratios of eyes with DME or PDR were much higher in the current study of all Asian participants from a single center. We excluded eyes with SRD, poor signal strength, or motion artifacts. Future multicenter prospective studies should be planned to confirm the reproducibility. The second limitation was that we used a specific SS-OCTA machine, although the signal strength and interscan time in each OCTA device influence image quality of vessels including microaneurysms and dilated vessels. Finally, we employed a specific procedure for the creation of the outer en face images and subsequent image processing. In particular, there are several methods to remove the projection artifacts, although we just used one method provided by the manufacturer’s software. Further studies should show the generalizability for these points.

In conclusion, we defined the *dilated deep capillaries* on the *outer* en face OCTA images in DR. The quantitative index reflecting these lesions may be designated as a biomarker of vision-threatening DR and could shed light on the pathogenesis in the deep vascular plexus in DR.

## Data Availability

The raw data are provided by the corresponding author upon reasonable request.
